# *De novo* and comparative transcriptome analysis of cultivated and wild spinach

**DOI:** 10.1038/srep17706

**Published:** 2015-12-04

**Authors:** Chenxi Xu, Chen Jiao, Yi Zheng, Honghe Sun, Wenli Liu, Xiaofeng Cai, Xiaoli Wang, Shuang Liu, Yimin Xu, Beiquan Mou, Shaojun Dai, Zhangjun Fei, Quanhua Wang

**Affiliations:** 1 Development and Collaborative Innovation Center of Plant Germplasm Resources, College of Life and Environmental Sciences, Shanghai Normal University, Shanghai, 200234, China; 2 Boyce Thompson Institute for Plant Research, Cornell University, Ithaca, NY 14853, USA; 3 State Key Laboratory of Crop Stress Biology in Arid Areas, College of Horticulture, Northwest A&F University, Yangling, Shaanxi 712100, China; 4 National Engineering Research Center for Vegetables, Key Laboratory of Biology and Genetic Improvement of Horticultural Crops (North China), Beijing 100097, China; 5 USDA, Agricultural Research Station, 1636 E. Alisal Street, Salinas, CA 93905, USA; 6 USDA-ARS, Robert W. Holley Center for Agriculture and Health, Ithaca, NY 14853, USA

## Abstract

Spinach (*Spinacia oleracea* L.) is an economically important green leafy vegetable crop. In this study, we performed deep transcriptome sequencing for nine spinach accessions: three from cultivated S. *oleracea*, three from wild *S. turkestanica* and three from wild *S. tetrandra*. A total of approximately 100 million high-quality reads were generated, which were *de novo* assembled into 72,151 unigenes with a total length of 46.5 Mb. By comparing sequences of these unigenes against different protein databases, nearly 60% of them were annotated and 50% could be assigned with Gene Ontology terms. A total of 387 metabolic pathways were predicted from the assembled spinach unigenes. From the transcriptome sequencing data, we were able to identify a total of ~320,000 high-quality single nucleotide polymorphisms (SNPs). Phylogenetic analyses using SNPs as well as gene expression profiles indicated that *S. turkestanica* was more closely related to the cultivated *S*. *oleracea* than *S. tetrandra*. A large number of genes involved in responses to biotic and abiotic stresses were found to be differentially expressed between the cultivated and wild spinach. Finally, an interactive online database (http://www.spinachbase.org) was developed to allow the research community to efficiently retrieve, query, mine and analyze our transcriptome dataset.

Spinach (*Spinacia oleracea *L.) is an annual or biennial plant which belongs to the family Amaranthaceae. It is widely cultivated as an economically important green leafy vegetable crop for fresh consumption and processing^1^. The annual worldwide gross production of spinach in 2013 was approximately 23 million tonnes, of which around 91% was produced in China (FAOSTAT; http://faostat3.fao.org). Spinach is a rich source of iron, lutein, folate, vitamins, minerals, and antioxidants (USDA Nutrient Database; http://ndb.nal.usda.gov/ndb/search/list). Currently the major aims of spinach breeding programs are to develop varieties with traits including increased disease resistance (particularly against *Peronospora farinosa* downy mildew) and abiotic stress tolerance, late bolting, and improved yield and quality such as decreased levels of nitrate and oxalate, and increased levels of folate in spinach leaves^2^. Several markers linked to downy mildew resistance^3^ and sex determination^4^,^5^,^6^ have been developed. Efforts, although limited, have also been taken toward cloning genes of interest^7^,^8^,^9^ and functions of a few genes involved in stress responses have been characterized using transgenic approaches^10^. Despite considerable progress in the genetic improvement of spinach, it is still difficult to develop varieties with desirable traits, mainly due to the very limited genomic and genetic resources currently available for spinach.

Spinach is a diploid species (2n = 2x = 12)^4^, with an estimated genome size of 989 Mb^11^. Currently, there are only 225 spinach expressed sequenced tags (ESTs) and 1,053 nucleotide sequences, among which the vast majority are chloroplast genome sequences, that are publicly available in GenBank. This leads to very limited molecular markers in spinach that are tightly linked with interesting traits. Recently the genome of sugar beet (*Beta vulgaris ssp. vulgaris*), another species in the Amaranthaceae family, has been reported^12^. For the purpose of comparative genomics and evolutionary analysis, Dohm *et al.*^12^ also generated a draft genome of a cultivated spinach, which was recently annotated^13^. Although the assembly represents only half of the spinach genome and contains many short assembled fragments, it contains the majority of the transcribed region^13^ and provides a valuable resource for spinach research and breeding. Furthermore, a more comprehensive spinach genome assembly is being generated (https://pag.confex.com/pag/xxiii/webprogram/Paper16426.html), providing additional valuable resource for spinach.

In spinach, two known wild species *S. turkestanica* Iljin and *S. tetrandra* Stev. have been documented. The two wild species are found to be distributed over western parts of Asia, *S. turkistanica* in Turkmenistan, Uzbekistan, and Kazakhstan, and *S. tetrandra* in the Caucasus area, in Armenia and Kurdistan between Iran, Iraq, and Turkey^13^. The exact origin of the cultivated spinach is still unknown. The geographical distribution of these wild species and the generally high sexual compatibility with cultivated *S. oleracea* suggest that cultivated spinach may have originated through the domestication of one or both of the wild species^14^. The wild *S. tetrandra* and *S. turkestanica* have been used as parents to construct genetically broad segregating offspring populations which have been further used to construct genetic maps and to map genetic factors determining dioecious sex expression in spinach^4^,^5^,^6^. In addition, the two wild species have already proven to be valuable sources of different kinds of disease resistances^15^,^16^,^17^. However, so far, exploring the wild relatives for spinach improvement has been limited and the genetic structure of spinach germplasm remains largely unknown. Thus, developing genomic resources of spinach and further research on the genetic diversity and phylogenetic relationship of the spinach germplasm will provide valuable information that can be used for better germplasm utilization and for facilitating breeding of new spinach varieties.

In this study, we report the transcriptome characterization of cultivated and wild spinach using the high-throughput Illumina sequencing technology. Strand-specific RNA-Seq libraries were constructed and sequenced for a total of nine spinach accessions including three from cultivated *S. oleracea*, three from wild *S. tetrandra* and three from wild *S. turkestanica*. The high-quality Illumina reads were *de novo* assembled into unique transcripts, which were then extensively evaluated and annotated. Single nucleotide polymorphisms (SNPs) and differentially expressed genes among the nine spinach accessions were identified and phylogenetic relationship and genetic diversity of cultivated and wild spinach were inferred. Our transcriptome data provide a valuable resource for future functional studies and marker assisted breeding in spinach.

## Results and Discussion

### Transcriptome sequencing and *de novo* assembly

We constructed strand-specific RNA-Seq libraries from the entire seedlings of nine different spinach accessions, including three from cultivated *S. oleracea*, Sp78 (S13-32), Sp82 (JQSZ13-3) and Sp90 (JQ13-1), three from wild *S. turkestanica*, Sp49 (PI 647864), Sp50 (PI 647865) and Sp51 (PI 662295), and three from wild *S. tetrandra,* Sp40 (PI 608712), Sp42 (PI 647860) and Sp43 (PI 647861). These libraries were sequenced on an Illumina HiSeq 2000 system; and a total of 104,377,466 reads with length of 101 bp were obtained. After removing adaptor and low quality sequences, as well as reads from ribosomal RNA (rRNA) contaminations, we obtained a total of 99,282,817 high-quality cleaned reads, consisting of 9,648,869,918 nucleotides, with at least 8 million reads for each accession (Table 1).

**Figure 1:**
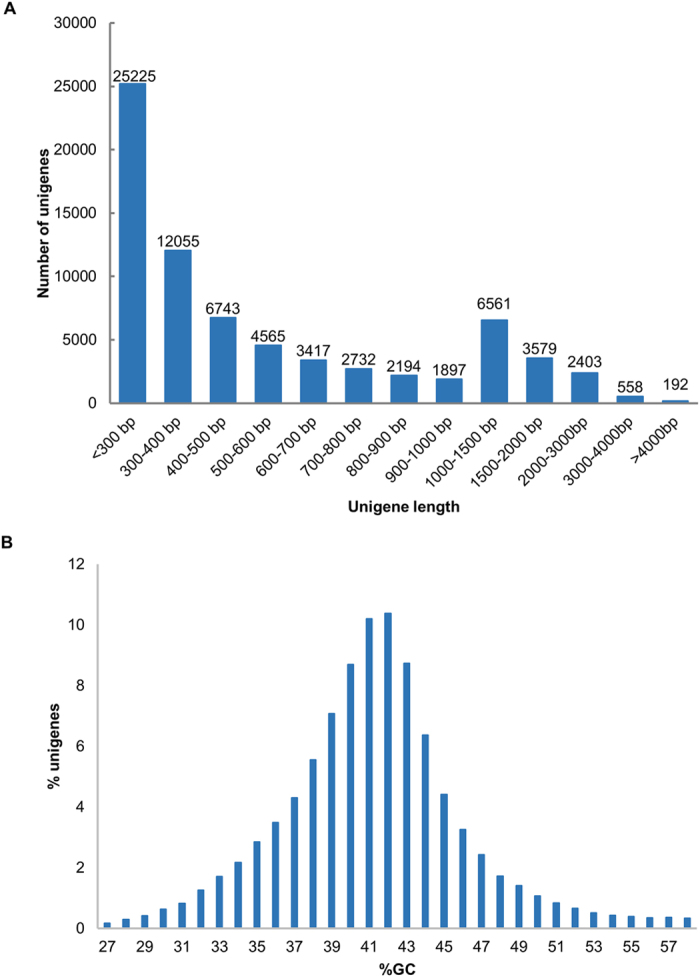
Length (A) and GC content (B) distribution of spinach unigenes.

These high-quality cleaned sequences were then *de novo* assembled into unique transcripts (unigenes). A total of 72,151 assembled unigenes were obtained, with an average length of 644 bp and N50 length of 974 bp. The assembled transcriptome was approximately 46.5 Mb in size. The length distribution of the assembled unigenes is shown in Fig. 1A. Although most unigenes were short, we did assemble approximately 13,300 unigenes that were longer than 1,000 bp; the majority of which could be full length transcripts. The GC content of the assembled spinach unigenes was 42.5% and its distribution peaked at around 42% (Fig. 1B), which was comparable to the GC content of Arabidopsis transcripts (42.3%; TAIR version 10 cDNA).

We then mapped the assembled unigenes to the draft spinach genome assembly^12^. Using a cutoff of at least 95% sequence identity and 90% coverage, a total of 53,130 (73.6%) unigenes could be mapped to the genome assembly. We further compared the spinach unigene sequences to the annotated spinach gene set^13^. A total of 18,447 (85%) out of 21,703 spinach predicted genes matched the unigenes, among which 17,517 (95%) matched with greater than 98% nucleotide identity. However, among the 72,151 unigenes, only 42,952 (59.5%) matched the spinach predicted genes, among which 40,082 (93.3%) matched with greater than 98% nucleotide identity. The high coverage of the spinach gene set by our unigenes indicates the broad representation of our unigenes. The relatively low coverage of our unigenes by the draft genome and the gene set could be due to incompleteness of the genome assembly, and the presence of large amount of non-coding RNAs, novel genes not predicted in the genome and highly divergent unigene sequences from wild species etc., suggesting our unigene set can serve as a valuable complementary resource for spinach genomics and functional genomics.

We further checked the quality of our assembled spinach unigenes by comparing their sequences to a core set of eukaryotic genes using BUSCO^18^. The result revealed that 69.9% of BUSCO genes were “complete”, 13.5% were “fragmented”, and the remaining 16.6% were “missing”. The quality of our assembled spinach unigenes was comparable to or better than that of the majority of transcriptome assemblies listed in Simao *et al.*^18^.

### Functional annotation of spinach transcriptome

To functionally annotate the spinach assembled unigenes, we compared their sequences against different protein databases including TrEMBL, Swiss-Prot and Arabidopsis TAIR10 using BLASTX. Our analysis revealed that 40,347 (55.9%), 28,580 (39.6%), and 35,461 (49.1%) spinach unigenes matched known proteins in TrEMBL, Swiss-Prot, and Arabidopsis, respectively. Based on the BLASTX results against these three databases, we were able to assign human readable functional descriptions for 42,019 (58.2%) spinach unigenes using the AHRD pipeline (https://github.com/asishallab/AHRD-1). Based on these annotations, we identified a total of 573 (0.8%) expressed transposable elements. It is worth noting that a relatively large portion of spinach unigenes had no hits to any known proteins. This is not unexpected as these unigenes could represent long non-coding RNAs, which were recently found highly abundant in plants such as Arabidopsis^19^, rice^20^ and maize^21^. These unigenes could also be spinach specific genes, as well as short fragments mainly from the untranslated (e.g 5′ and 3′ UTR) or non-conserved regions of protein-coding transcripts.

We then compared the protein sequences predicted from the genome of sugar beet (version 1.2)^12^, a species in the same Amaranthaceae family as spinach, against the spinach unigenes. A total of 21,296 out of 26,923 (79%) sugar beet proteins had hits to 42,526 spinach unigenes. Same analysis performed on Arabidopsis proteins revealed that 22,897 out of 27,416 (83.5%) proteins had hits to 36,466 spinach unigenes. The higher percentage for Arabidopsis proteins could be mainly due to the better annotation of the Arabidopsis genome. Nevertheless, the high percentage of proteins in both sugar beet and Arabidopsis covered by spinach unigenes further suggested the broad representation of spinach genes by our assembled unigene set.

### Gene ontology and metabolic pathways

We further assigned Gene Ontology (GO) terms to spinach unigenes. A total of 34,522 unigenes (47.8%) could be assigned with at least one GO term, among which 29,277 were assigned with at least one GO term in the biological process category, 29,972 in the molecular function category and 28,074 in the cellular component category; while 22,808 (31.6%) spinach unigenes were annotated with GO terms from all the three categories. Using a set of plant-specific GO slims (http://www.geneontology.org/GO.slims.shtml), the spinach unigenes were classified into different functional categories (Supplementary Table S1). Within the biological process category, cellular process, response to stress, biosynthetic process, nucleobase-containing compound metabolic process, and cellular component organization were among the most highly represented groups (Supplementary Figure 1A). Within the molecular function category, the top five most abundant groups were binding, nucleotide binding, hydrolase activity, catalytic activity and protein binding (Supplementary Figure 1B). Membrane, nucleus, plasma membrane, cytoplasm, and cytosol were the most represented groups within the cellular component category (Supplementary Figure 1C). It is worth noting that GO annotations also revealed a large number of genes involved in some important biological processes such as signal transduction, secondary metabolism and cell differentiation (Supplementary Table S1).

To further demonstrate the usefulness of spinach assembled unigenes, we predicted metabolic pathways represented by these unigenes. A total of 387 pathways represented by a total of 2,785 unigenes were predicted. These pathways represent the majority of plant metabolic pathways for compound biosynthesis, degradation, utilization, and assimilation, and pathways involved in the processes of detoxification and generation of precursor metabolites and energy (Supplementary Table S2). As expected, the most highly represented pathways included those related to photosynthesis and respiration. Spinach contains abundant active antioxidant components and folate^22^. The biosynthesis pathways of secondary metabolite were well represented, including those involved in folate biosynthesis and transformations and flavonoid biosynthesis (Supplementary Table S2).

### SNP identification and phylogenetic analysis of the nine spinach accessions

SNPs are currently the most widely used molecular markers as they are hypervariable, multiallelic, codominant, locus-specific, and are evenly distributed throughout the genome. They represent a valuable resource to facilitate candidate gene or quantitative trait locus (QTL) identification, and population structure and evolutionary analysis, and to accelerate plant breeding through marker assisted selection. For SNPs derived from transcriptome sequences, they are directly linked to expressed genes. In this study, a total of 319,838 SNPs were identified in the transcribed regions of the nine spinach accessions, of which 76,433 had genotype information for all nine spinach accessions and were homozygous. These SNPs provided a valuable resource for genetic linkage mapping and marker-assisted breeding, as well as the analysis of interesting traits in spinach and closely related species.

**Figure 2:**
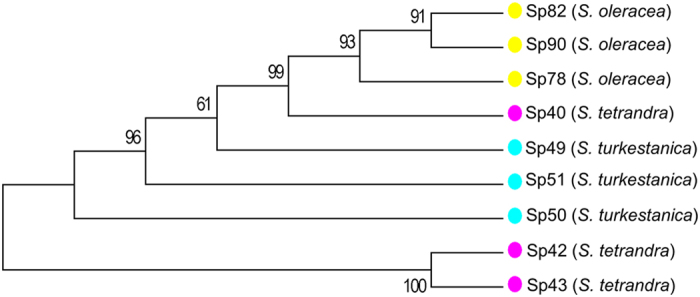
Neighbor-joining phylogenetic tree of spinach accessions on the basis of SNPs.

The 76,433 homozygous SNPs were then used to construct a maximum-likelihood phylogenetic tree of the nine spinach accessions. The tree revealed that one *S. tetrandra* accession, Sp40 (PI 608712), was unexpectedly clustered together with *S. oleracea* and *S. turkestanica* accessions and far away from the other two *S. tetrandra* accessions (Fig. 2). The global expression profile analysis further supported this phylogenetic relationship (see the section below). This indicates that PI 608712 could be from an error in the germplasm documentation system or during the sample handling; therefore we excluded this accession from further SNP analysis.

The phylogenetic analysis supports that cultivated spinach (*S. oleracea*) are more closely related to *S. turkestanica* than to *S. tetrandra*. Among the eight spinach accessions (after excluding PI 608712), we identified a total of 76,352 SNPs that had genotype information in all the eight accessions. Of these SNPs, 565, 1,143, and 3,124 were identified in the three cultivated *S. oleracea*, the three *S. turkestanica*, and the two *S. tetrandra* accessions, respectively; and 1,690, 75,599, 75,924 were identified in the six *S. oleracea* and *S. turkestanica*, the five *S. oleracea* and *S. tetrandra*, and the five *S. turkestanica* and *S. tetrandra* accessions, respectively (Table 2). This indicates that as expected, the cultivated spinach has much less genetic diversity than the wild. It also provides further evidence to support that *S. turkestanica* is much more closely related to the cultivated spinach than *S. tetrandra*, implying that *S. turkestanica* could be the direct wild progenitor of the cultivated spinach. Further studies, such as genome survey of a larger collection of different cultivated and wild spinach, would provide a clearer picture of spinach evolution and domestication.

### Comparative transcriptome analysis of wild and cultivated spinach

Using the RNA-Seq data, we derived gene expression profiles in all nine spinach accessions (Supplementary Table S3). Both Pearson correlation coefficients (Supplementary Table S4) and hierarchical clustering analysis (Supplementary Figure S2) of gene expression profiles indicated that Sp40, which was originally assigned to *S. tetrandra* in the GRIN National Genetic Resources Program database (http://www.ars-grin.gov/), was more closely related to *S. oleracea* and *S. turkestanica* than *S. tetrandra*, consistent with the phylogenetic analysis result based on SNPs. We therefore excluded Sp40 in our downstream expression analysis.

**Figure 3:**
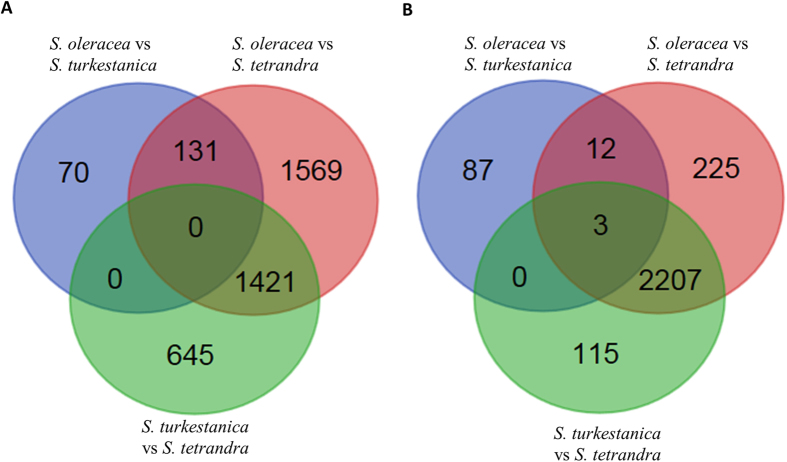
Venn diagram showing the number of genes which displayed higher (A) or lower (B) expression in three different comparisons: *S. oleracea* vs *S. turkestanica*, *S. oleracea* vs *S. tetrandra*, and *S. turkestanica* vs *S. tetrandra*.

We then identified highly differentially expressed genes between any two of the three spinach species. We defined highly differentially expressed genes as those with 1) the minimum expression levels in one species being at least five times or higher of the maximum expression levels in the other species, 2) the minimum expression levels in the higher expressed species being at least five RPKM (reads per kilobase of exon model per million mapped reads), and 3) false discovery rates less than 0.05. We identified a total of 201 and 3,121 unigenes that had higher expression levels in *S. oleracea* than in *S. turkestanica* and in *S. tetrandra*, respectively, and 2,066 unigenes that had higher expression levels in *S. turkestanica* than in *S. tetrandra* (Supplementary Table S3). We also identified a total of 102 and 2,447 unigenes that had lower expression levels in *S. oleracea* than in *S. turkestanica* and in *S. tetrandra*, respectively, and 2,325 unigenes that had lower expression levels in *S. turkestanica* than in *S. tetrandra* (Supplementary Table S3). A large number of unigenes showing higher or lower expression in *S. oleracea* than in *S. tetrandra* were also expressed in higher or lower levels in *S. turkestanica* than in *S. tetrandra* (Fig. 3). GO term enrichment analysis indicated that genes involved in various biological processes such as responses to different abiotic stresses and hormones, defense response, and cell wall assembly and organization were significantly enriched in genes showing higher expression in *S. oleracea* than in *S. turkestanica*; while those involved in the regulation of seed germination, post-embryonic development and flower development, and in the responses to different abiotic stresses were significantly enriched in genes showing lower expression in *S. oleracea* than in *S. turkestanica* (Supplementary Table S5). The fact that a large number of genes involved in abiotic stress responses showed differential expression between cultivated *S. oleracea* and wild *S. turkestanica* is consistent with the observations that wild plants are generally more tolerant to many abiotic stresses than cultivated species^23^,^24^. We also found that genes involved in the metabolic processes of several secondary metabolites such as cinnamic acid ester, triterpenoid and anthocyanin were significantly enriched in genes showing differential expression between *S. oleracea* and *S. tetrandra*. This is consistent with the received wisdom that during domestication of crop plants, the contents of some secondary metabolites have been reduced^25^,^26^. This also suggests that wild spinaches are valuable materials that can be used to breed spinaches with higher contents of secondary metabolites, therefore increasing their nutritional values and resistances to biotic and abiotic stresses.

### Database for spinach genomics

To allow the research and breeding community to efficiently retrieve, mine and analyze our spinach transcriptome data, we developed an interactive online database, SpinachBase. SpinachBase is publicly available and can be accessed at http://www.spinachbase.org. Our ultimate goal is to make SpinachBase a one-stop-shop for spinach genomics. Currently the database is designed to allow users to easily retrieve and visualize the spinach transcriptome sequence and expression profile data through a set of query interfaces and analysis tools. The database includes a BLAST tool which allows users to compare specific sequences against the assembled spinach unigene sequences, and unigene query interfaces which gives detailed information of a specific spinach unigene including its sequences, functional annotations and expression profiles in the nine spinach accessions. A database containing spinach metabolic pathways (SpinachCyc) predicted from spinach unigene sequences is also included in SpinachBase.

## Materials and Methods

### Plant material

Seeds of the three *S. turkestanica* and the three *S. tetrandra* accessions were obtained from the North Central Regional Plant Introduction Station, Ames, Iowa and seeds of the three cultivated spinach varieties were provided by Laizhou Seed Company (Shandong, China) and Jiuquan Suzhou Seed company (Gansu, China). Both wild and cultivated spinach plants were grown under standard greenhouse conditions with a 16-hour light (27 °C) and 8-hour dark (19 °C) cycle. The entire 20-day-old plants were collected for each accession, immediately frozen in liquid nitrogen and stored at −80 °C till use.

### Strand-specific RNA-Seq library construction and sequencing

Total RNA was extracted using the QIAGEN RNeasy Plant Mini Kit following the manufacturer’s instructions. The quality and quantity of RNA were assessed by electrophoresis on 1% agarose gels and by a NanoDrop 1000 spectrophotometer (Thermo Scientific, USA), respectively. Strand-specific RNA-Seq libraries were constructed using the protocol described in Zhong *et al.*^27^ and sequenced on an Illumina HiSeq 2000 platform using the single-end mode with the read length of 101 bp. The raw sequencing data has been deposited in NBCI sequence read archive (SRA) under the accession numbers SRP052589, SRP052590 and SRP052591.

### RNA-Seq data processing, *de novo* assembly and unigene alignment

RNA-Seq raw reads were first processed using Trimmomatic^28^ to remove adaptor and low quality sequences. Reads shorter than 40 bp were discarded. The resulting reads were then aligned to the ribosomal RNA database^29^ using bowtie^30^ and those that could be aligned were discarded. The resulting high-quality cleaned reads from the nine accessions were *de novo* assembled into contigs using Trinity^31^ with the minimum kmer coverage set to 2. Following assembly, the high-quality cleaned reads were then aligned back to assembled contigs using Bowtie^30^ allowing up to two mismatches and only the best alignments of each read were retained. Then for each contig, the numbers of reads aligned in sense and antisense directions, respectively, were derived. To remove false transcripts with antisense direction which were due to the incomplete digestion of the 2^nd^ strand during the strand-specific RNA-Seq library construction^27^, contigs with the number of reads aligned in sense direction less than 1/10 of the number of reads aligned in antisense direction were discarded. The resulting assembled contigs were then blasted against GenBank Nucleotide (nt) database and those having hits only to sequences from viruses, bacteria, and archaea were discarded. Next, the rRNA, low-complexity, and polyA/T sequences were removed or trimmed from the contigs using SeqClean (http://sourceforge.net/projects/seqclean/). The remaining contigs were further clustered to remove redundancies and assembled into the final unigene set using iAssembler^32^ with sequence identity cutoff set to 97%. The assembled unigenes were aligned to the spinach genome assembly^12^ using SPALN^33^. The unigene sequences were also compared with the spinach predicted gene set^13^ using MEGABLAST with a cutoff of percent identity greater than 95, E value less than 1e-30, and alignment length greater than 100 bp.

### Functional annotation of spinach assembled unigenes

The final assembled spinach unigenes were blasted against the UniProt (Swiss-Prot and TrEMBL)^34^, Arabidopsis protein (version TAIR10)^35^ and sugar beet protein^12^ databases with a cutoff E-value of 1e-5. Based on the UniProt and Arabidopsis protein blast results, functional descriptions (human readable descriptions) were assigned to each spinach unigene using AHRD (https://github.com/asishallab/AHRD-1). Gene ontology (GO) terms were assigned to the spinach assembled unigenes based on the GO terms annotated to their corresponding homologues in the UniProt database^36^. Metabolic pathways were predicted from the spinach unigenes using the Pathway Tools^37^.

### SNP identification and phylogenetic relationship analysis

High-quality cleaned RNA-seq reads from each accession were aligned back to the final assembled spinach unigenes using BWA^38^. To minimize the artifacts of PCR amplification, only one of the duplicated RNA-Seq reads was used for mapping. Based on the alignments, genotypes at each contig position were inferred for each accession based on the mpileup files generated by SAMtools^39^. SNPs were then identified based on the genotype information, and the identified SNPs were supported by at least three distinct RNA-Seq reads and had an allele frequency > 75%. SNPs which had information in all nine spinach accessions were then used to construct a maximum-likelihood tree using MEGA5 (ref. 40) with 1000 bootstraps.

### RNA-Seq gene expression analysis

High-quality cleaned reads were aligned to spinach unigenes using Bowtie^30^ allowing up to two mismatches. Only the best alignments for each read were retained. Following alignments, raw read count for each spinach gene in each sample was derived and normalized to RPKM. The expression profile data of the nine spinach accessions were hierarchically clustered using the average linkage clustering method implemented in the BCLUST program^41^. The Pearson correlation coefficient was used to measure the similarity of the profiles in the clustering. The robustness of the clustering trees was tested using the bootstrap method implemented in BCLUST with 1000 replicates. The clustering tree was drawn using the MEGA5 program^40^. The significance of differential unigene expression among the three spinach species was determined using edgeR^42^, and raw p-values of multiple tests were corrected using false discovery rate^43^ (FDR). GO terms enriched in differentially expressed genes were identified using GO::TermFinder^44^.

## Additional Information

**How to cite this article**: Xu, C. *et al.* *De novo* and comparative transcriptome analysis of cultivated and wild spinach. *Sci. Rep.* **5**, 17706; doi: 10.1038/srep17706 (2015).

## Supplementary Material

Supplementary Information

Supplementary Table S2

Supplementary Table S3

## Figures and Tables

**Table 1:** Summary of spinach transcriptome sequences.

sample ID	Accession No.	Species	No. raw reads	No. high quality reads^a^	No. rRNA reads	No. final cleaned reads^b^	Final cleaned nucleotides
Sp40	PI 608712	*S. tetrandra*	11,220,670	10,823,210	130,619	10,692,591	1,037,741,475
Sp42	PI 647860	*S. tetrandra*	9,301,545	9,035,669	128,237	8,907,432	859,510,808
Sp43	PI 647861	*S. tetrandra*	11,968,303	11,705,229	255,255	11,449,974	1,117,491,750
Sp49	PI 647864	*S. turkestanica*	9,580,222	9,126,379	54,604	9,071,775	872,975,381
Sp50	PI 647865	*S. turkestanica*	15,485,184	15,125,268	331,496	14,793,772	1,438,908,213
Sp51	PI 662295	*S. turkestanica*	16,640,543	16,276,742	401,807	15,874,935	1,547,778,458
Sp78	S13–32	*S. oleracea*	11,174,738	10,645,123	93,995	10,551,128	1,025,072,557
Sp82	JQSZ13-3	*S. oleracea*	10,560,339	10,039,569	154,651	9,884,918	959,870,221
Sp90	JQ13-1	*S. oleracea*	8,445,922	8,255,054	198,762	8,056,292	789,521,055
Total			104,377,466	101,032,243	1,749,426	99,282,817	9,648,869,918

^a^Reads left after removing adaptor and low quality sequences.

^b^Reads left after removing rRNA reads from the high quality reads.

**Table 2:** Summary of SNPs identified in spinach.

category	No. SNPs
Three *S. oleracea* accessions	565
Three *S. turkestanica* accessions	1,143
Two *S. tetrandra* accessions (excluding Sp40)	3,124
Three *S. oleracea* accessions and three *S. turkestanica* accessions	1,690
Three *S. oleracea* accessions and two *S. tetrandra* accessions	75,599
Three *S. turkestanica* accessions and two *S. tetrandra* accessions	75,924
All eight accessions (excluding Sp40)	76,352
All nine accessions	76,433

Out of 319,838 SNPs identified in spinach, only 76,433 homozygous SNPs with genotype information in all nine accessions were included in the summary.
