# Supplementary material for: De novo and comparative transcriptome analysis of cultivated and wild spinach
Source: Sci Rep. 2015 Dec 4;5:17706. doi: 10.1038/srep17706 (PMC4669492; doi:10.1038/srep17706)
Supplement: Supplementary Information [file srep17706-s1.pdf]

Supplementary Information

De novo and comparative transcriptome analysis of cultivated and wild spinach

Chenxi Xu[1*], Chen Jiao[2,3*], Yi Zheng[2], Honghe Sun[2,4], Wenli Liu[2], Xiaofeng Cai[1], Xiaoli Wang[1], Shuang Liu[1], Yimin Xu[2], Beiquan Mou[5], Shaojun Dai[1], Zhangjun Fei[2,6], Quanhua Wang[1]

[1]Development and Collaborative Innovation Center of Plant Germplasm Resources, College of Life and Environmental Sciences, Shanghai Normal University, Shanghai, 200234, China

[2]Boyce Thompson Institute for Plant Research, Cornell University, Ithaca, NY 14853, USA

[3]State Key Laboratory of Crop Stress Biology in Arid Areas, College of Horticulture, Northwest A&F University, Yangling, Shaanxi 712100, China

[4]National Engineering Research Center for Vegetables, Key Laboratory of Biology and Genetic Improvement of Horticultural Crops (North China), Beijing 100097, China.

[5]USDA, Agricultural Research Station, 1636 E. Alisal Street, Salinas, CA 93905, USA

[6]USDA-ARS, Robert W. Holley Center for Agriculture and Health, Ithaca, NY 14853, USA

[*]These authors contributed equally to this work

Correspondence and requests for materials should be addressed to Q.W. (email: wangquanhua@shnu.edu.cn) or Z.F. (email: zf25@cornell.edu)

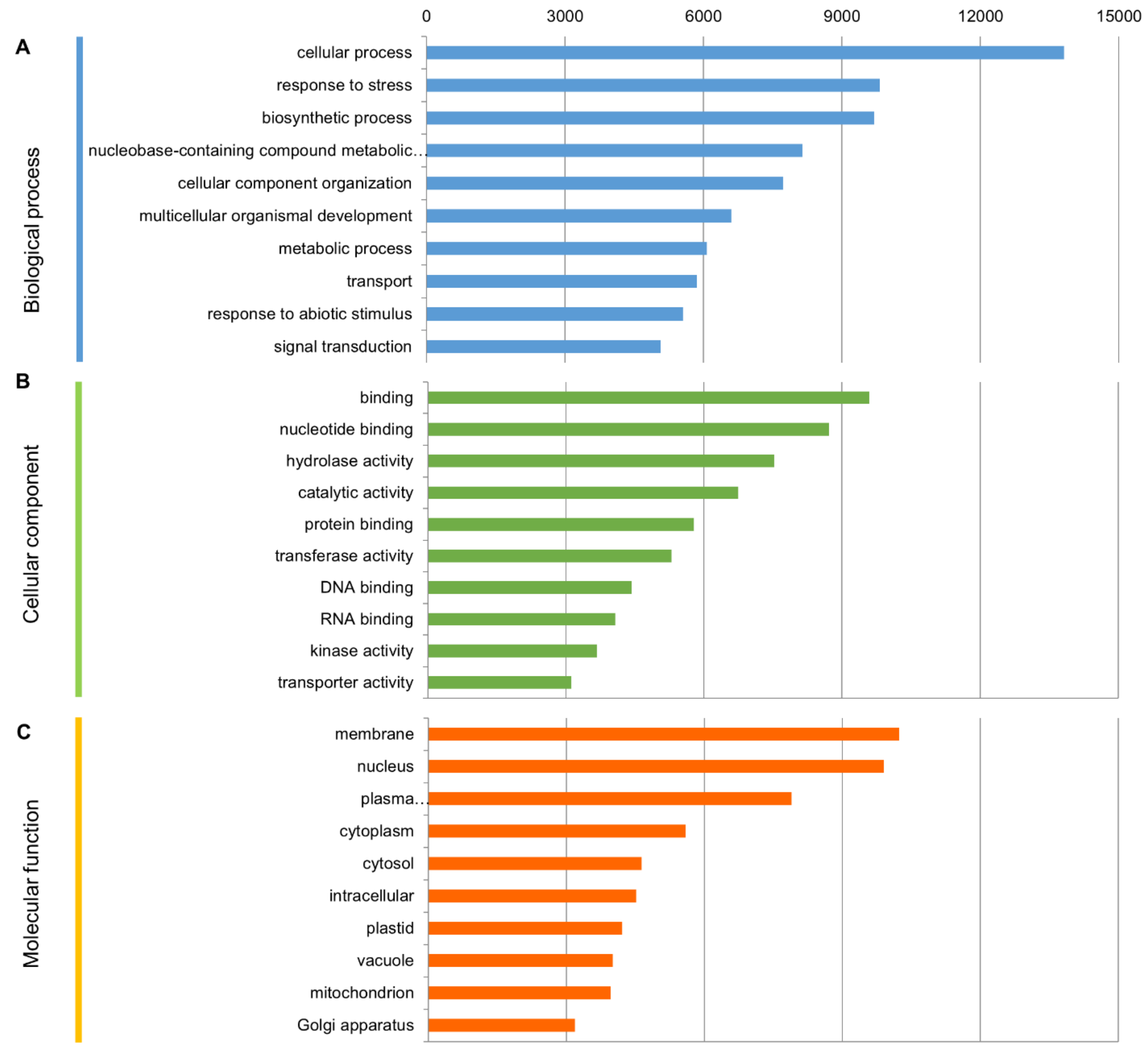

Supplementary Figure S1. Functional classification of spinach unigenes.

Spinach unigenes were functionally classified into different groups based on a set of plant specific GO Slims. The top ten GO slims in the category of biological process (A), cellular component (B) and molecular function (C) are shown.

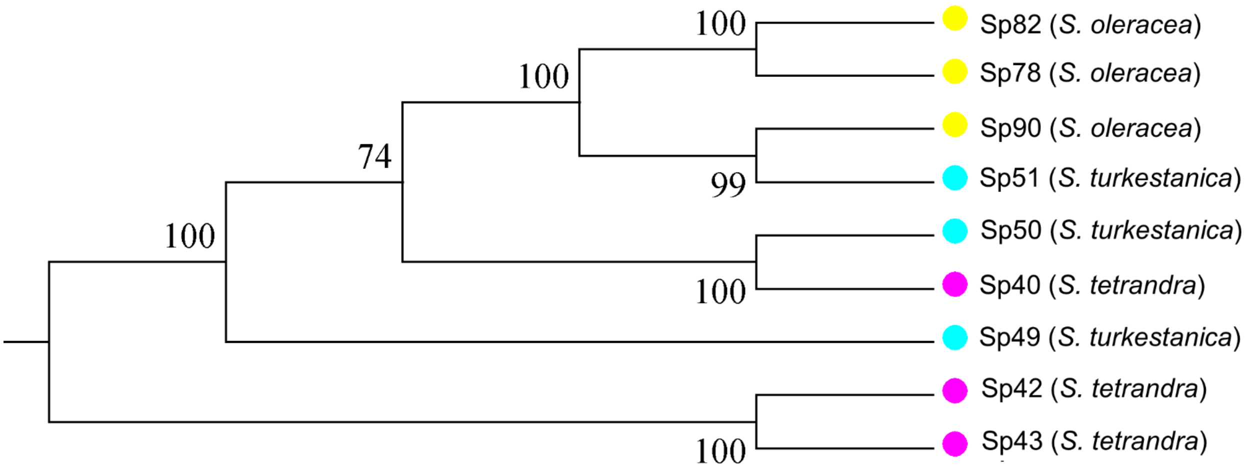

Supplementary Figure S2. Phylogenetic tree of the nine spinach accessions based on gene expression profiles.

Supplementary Table S1. GO classification of assembled spinach unigenes.

GO ID	GO term	category	No. unigenes
GO:0000003	reproduction	biological process	4717
GO:0005975	carbohydrate metabolic process	biological process	2846
GO:0006091	generation of precursor metabolites and energy	biological process	946
GO:0006139	nucleobase-containing compound metabolic process	biological process	8139
GO:0006259	DNA metabolic process	biological process	3386
GO:0006412	translation	biological process	1129
GO:0006464	cellular protein modification process	biological process	4339
GO:0006629	lipid metabolic process	biological process	2668
GO:0006810	transport	biological process	5861
GO:0006950	response to stress	biological process	9817
GO:0007049	cell cycle	biological process	3395
GO:0007154	cell communication	biological process	1237
GO:0007165	signal transduction	biological process	5074
GO:0007267	cell-cell signaling	biological process	561
GO:0007275	multicellular organismal development	biological process	6598
GO:0007610	behavior	biological process	501
GO:0008152	metabolic process	biological process	6067
GO:0008219	cell death	biological process	2346
GO:0009056	catabolic process	biological process	4955
GO:0009058	biosynthetic process	biological process	9697
GO:0009605	response to external stimulus	biological process	4762
GO:0009606	tropism	biological process	317
GO:0009607	response to biotic stimulus	biological process	4004
GO:0009628	response to abiotic stimulus	biological process	5553
GO:0009653	anatomical structure morphogenesis	biological process	4391
GO:0009719	response to endogenous stimulus	biological process	4908
GO:0009790	embryo development	biological process	2594
GO:0009791	post-embryonic development	biological process	3616
GO:0009835	fruit ripening	biological process	46
GO:0009838	abscission	biological process	183
GO:0009856	pollination	biological process	681
GO:0009875	pollen-pistil interaction	biological process	151
GO:0009908	flower development	biological process	1957
GO:0009987	cellular process	biological process	13807
GO:0009991	response to extracellular stimulus	biological process	1676
GO:0015979	photosynthesis	biological process	426
GO:0016043	cellular component organization	biological process	7723
GO:0016049	cell growth	biological process	1641

GO:0019538	protein metabolic process	biological process	2323
GO:0019725	cellular homeostasis	biological process	1133
GO:0019748	secondary metabolic process	biological process	935
GO:0030154	cell differentiation	biological process	4273
GO:0040007	growth	biological process	1348
GO:0040029	regulation of gene expression, epigenetic	biological process	713
GO:0000166	nucleotide binding	molecular function	8715
GO:0003676	nucleic acid binding	molecular function	1941
GO:0003677	DNA binding	molecular function	4428
GO:0003682	chromatin binding	molecular function	866
GO:0003700	sequence-specific DNA binding transcription factor activity	molecular function	2014
GO:0003723	RNA binding	molecular function	4072
GO:0003774	motor activity	molecular function	375
GO:0003824	catalytic activity	molecular function	6742
GO:0004518	nuclease activity	molecular function	1367
GO:0004871	signal transducer activity	molecular function	1778
GO:0004872	receptor activity	molecular function	1217
GO:0005102	receptor binding	molecular function	815
GO:0005198	structural molecule activity	molecular function	1086
GO:0005215	transporter activity	molecular function	3123
GO:0005488	binding	molecular function	9587
GO:0005515	protein binding	molecular function	5780
GO:0008135	translation factor activity, nucleic acid binding	molecular function	365
GO:0008289	lipid binding	molecular function	877
GO:0016301	kinase activity	molecular function	3677
GO:0016740	transferase activity	molecular function	5297
GO:0016787	hydrolase activity	molecular function	7523
GO:0019825	oxygen binding	molecular function	31
GO:0030234	enzyme regulator activity	molecular function	884
GO:0030246	carbohydrate binding	molecular function	869
GO:0045182	translation regulator activity	molecular function	73
GO:0005576	extracellular region	cellular component	3108
GO:0005578	proteinaceous extracellular matrix	cellular component	130
GO:0005615	extracellular space	cellular component	260
GO:0005618	cell wall	cellular component	2033
GO:0005622	intracellular	cellular component	4516
GO:0005623	cell	cellular component	1655
GO:0005634	nucleus	cellular component	9900
GO:0005635	nuclear envelope	cellular component	612
GO:0005654	nucleoplasm	cellular component	2198

GO:0005730	nucleolus	cellular component	1786
GO:0005737	cytoplasm	cellular component	5586
GO:0005739	mitochondrion	cellular component	3957
GO:0005764	lysosome	cellular component	509
GO:0005768	endosome	cellular component	1499
GO:0005773	vacuole	cellular component	4003
GO:0005777	peroxisome	cellular component	663
GO:0005783	endoplasmic reticulum	cellular component	2246
GO:0005794	Golgi apparatus	cellular component	3185
GO:0005829	cytosol	cellular component	4633
GO:0005840	ribosome	cellular component	723
GO:0005856	cytoskeleton	cellular component	2016
GO:0005886	plasma membrane	cellular component	7891
GO:0009536	plastid	cellular component	4215
GO:0009579	thylakoid	cellular component	1042
GO:0016020	membrane	cellular component	10232
GO:0030312	external encapsulating structure	cellular component	31

Supplementary Table S2. Summary of biochemical pathways predicted from spinach unigenes.

pathway ID	pathway name	No. unigenes
PWY-5464	superpathway of cytosolic glycolysis (plants), pyruvate dehydrogenase and TCA cycle	132
PWY-6901	xylose degradation IV	98
ANAGLYCOLYSIS-PWY	glycolysis III (glucokinase)	97
GLUCONEO-PWY	gluconeogenesis I	96
GLYCOLYSIS	glycolysis I	93
TRNA-CHARGING-PWY	tRNA charging	88
PWY-1042	glycolysis IV (plant cytosol)	87
PWY-1081	homogalacturonan degradation	85
LIPASYN-PWY	phospholipases	69
PWY-5723	Rubisco shunt	67
PWY66-21	ethanol degradation II	67
PWY-735	jasmonic acid biosynthesis	64
PHOTOALL-PWY	oxygenic photosynthesis	62
PWY-6126	adenosine nucleotides de novo biosynthesis	62
PWY-6351	D-myo-inositol (1,4,5)-trisphosphate biosynthesis	62
PWY-6352	3-phosphoinositide biosynthesis	55
ETOH-ACETYLCOA-ANA-PWY	ethanol degradation I	54
PWY-4441	DIMBOA-glucoside degradation	54
CALVIN-PWY	Calvin-Benson-Bassham cycle	52
PWY-6629	superpathway of tryptophan biosynthesis	52
PWY-842	starch degradation I	52
PWY-6842	glutathione-mediated detoxification II	45
PWY-361	phenylpropanoid biosynthesis	44
PWY-6549	glutamine biosynthesis III	44
PWY-6773	callose biosynthesis	44
PWY-6803	phosphatidylcholine acyl editing	44
SUCSYN-PWY	sucrose biosynthesis	44
PWY0-162	pyrimidine ribonucleotides de novo biosynthesis	43
PWY-3781	aerobic respiration (cytochrome c)	43
PWY-622	starch biosynthesis	42
PWY-3561	choline biosynthesis III	41
TRESYN-PWY	trehalose biosynthesis I	39
PWY0-163	salvage pathways of pyrimidine ribonucleotides	37
PWY-181	photorespiration	37
PWY-5436	threonine degradation IV	37
BRANCHED-CHAIN-AA-SYN-PWY	superpathway of leucine, valine, and isoleucine biosynthesis	36
GLYCOGENSYNTH-PWY	glycogen biosynthesis I (from ADP-D-Glucose)	36
PWY-5121	superpathway of geranylgeranyldiphosphate biosynthesis II (via MEP)	36
PWY-5667	CDP-diacylglycerol biosynthesis I	36
PWY1F-FLAVSYN	flavonoid biosynthesis	35
PWY-5884	wax esters biosynthesis I	34
PWY-621	sucrose degradation III	33
TRIGLSYN-PWY	triacylglycerol biosynthesis	33
BGALACT-PWY	lactose degradation III	32
COLANSYN-PWY	colanic acid building blocks biosynthesis	32
PWY-2161	folate polyglutamylation	31
PWY-5690	TCA cycle II (eukaryotic)	31
PWY-1001	cellulose biosynthesis	30

PWY-241	C4 photosynthetic carbon assimilation cycle	30
PWY-6724	starch degradation II	30
PWY-5143	fatty acid activation	29
PWY-6348	phosphate acquisition	29
PWY-6357	phosphate utilization in cell wall regeneration	29
NONMEVIPP-PWY	methylerythritol phosphate pathway	28
PWY-3001	isoleucine biosynthesis I	28
PWY-5083	NAD/NADH phosphorylation and dephosphorylation	28
PWY-5829	geraniol and geranial biosynthesis	28
PWY-6527	stachyose degradation	28
TRPSYN-PWY	tryptophan biosynthesis	28
PENTOSE-P-PWY	pentose phosphate pathway	27
PWY-3821	galactose degradation III	27
PWY-2261	ascorbate glutathione cycle	26
PWY-5486	pyruvate fermentation to ethanol II	26
PWY-6333	acetaldehyde biosynthesis I	26
ETHYL-PWY	ethylene biosynthesis I (plants)	25
PWY-6317	galactose degradation I (Leloir pathway)	25
ARO-PWY	chorismate biosynthesis I	24
PWY0-1319	CDP-diacylglycerol biosynthesis II	24
PWY0-1334	NADH to cytochrome bd oxidase electron transfer	24
PWYQT-4429	CO2 fixation into oxaloacetate (anapleurotic)	24
SULFATE-CYS-PWY	superpathway of sulfate assimilation and cysteine biosynthesis	24
LEUSYN-PWY	leucine biosynthesis	22
PWY-5080	very long chain fatty acid biosynthesis	22
PWY-5686	uridine-5'-phosphate biosynthesis	22
GLYOXYLATE-BYPASS	glyoxylate cycle	21
PWY0-541	cyclopropane fatty acid (CFA) biosynthesis	21
PWY-2201	folate transformations I	21
PWY-3841	folate transformations II	21
PWY-5687	pyrimidine ribonucleotides interconversion	21
PWY-6577	farnesylcysteine salvage pathway	21
PWY-6596	adenosine nucleotides degradation I	21
MANNOSYL-CHITO-DOLICHOL-BIOSYNTHESIS	dolichyl-diphosphooligosaccharide biosynthesis	20
PWY-3282	superpathway of ammonia assimilation (plants)	20
PWY-4661	1D-myo-inositol hexakisphosphate biosynthesis III (Spirodela polyrrhiza)	20
PWY-5386	methylglyoxal degradation I	20
PWY-5787	oligomeric urushiol biosynthesis	20
PWY-5918	heme biosynthesis I	20
PWY-6964	ammonia assimilation cycle II	20
PWY-724	superpathway of lysine, threonine and methionine biosynthesis II	20
SER-GLYSYN-PWY	superpathway of serine and glycine biosynthesis I	20
PWY-2541	plant sterol biosynthesis	19
PWY-4081	glutathione redox reactions I	19
PWY-6963	ammonia assimilation cycle I	19
PWY-43	putrescine biosynthesis II	18
PWY-5381	pyridine nucleotide cycling (plants)	18
PWY-5910	superpathway of geranylgeranyldiphosphate biosynthesis I (via mevalonate)	18
PWY-6959	L-ascorbate degradation V	18
CYANCAT-PWY	cyanate degradation	17

DETOX1-PWY	superoxide radicals degradation	17
OXIDATIVEPENT-PWY	pentose phosphate pathway (oxidative branch)	17
PWY-5173	superpathway of acetyl-CoA biosynthesis	17
PWY-6164	3-dehydroquinate biosynthesis I	17
VALDEG-PWY	valine degradation I	17
FASYN-ELONG-PWY	fatty acid elongation -- saturated	16
HISTSYN-PWY	histidine biosynthesis	16
PWY-5973	cis-vaccenate biosynthesis	16
ARGSYNBSUB-PWY	arginine biosynthesis II (acetyl cycle)	15
GLYSYN-PWY	glycine biosynthesis I	15
PWY-5041	S-adenosyl-L-methionine cycle II	15
PWY-5053	superpathway of GA12 biosynthesis	15
PWY-5921	L-glutamine biosynthesis II (tRNA-dependent)	15
PWY-5971	palmitate biosynthesis II (bacteria and plants)	15
PWY-6305	putrescine biosynthesis IV	15
RIBOSYN2-PWY	flavin biosynthesis I (bacteria and plants)	15
SALVADEHYPOX-PWY	adenosine nucleotides degradation II	15
THRESYN-PWY	threonine biosynthesis	15
PWY-5791	1,4-dihydroxy-2-naphthoate biosynthesis II (plants)	14
PWY-6475	trans-lycopene biosynthesis II (plants)	14
PYRUVDEHYD-PWY	acetyl-CoA biosynthesis I (pyruvate dehydrogenase complex)	14
ARGDEG-V-PWY	arginine degradation X (arginine monooxygenase pathway)	13
ASPARAGINE-BIOSYNTHESIS	asparagine biosynthesis I	13
ASPARAGINESYN-PWY	asparagine biosynthesis II	13
CHLOROPHYLL-SYN	chlorophyllide a biosynthesis I	13
HEME-BIOSYNTHESIS-II	heme biosynthesis from uroporphyrinogen-III I	13
ILEUSYN-PWY	isoleucine biosynthesis I (from threonine)	13
PWY0-1299	arginine dependent acid resistance	13
PWY0-1313	acetate conversion to acetyl-CoA	13
PWY0-1325	superpathway of asparagine biosynthesis	13
PWY0-1353	succinate to cytochrome bd oxidase electron transfer	13
PWY-1121	suberin biosynthesis	13
PWY-5059	pinobanksin biosynthesis	13
PWY-5692	allantoin degradation to glyoxylate II	13
PWY-5698	allantoin degradation to ureidoglycolate II (ammonia producing)	13
PWY-6151	S-adenosyl-L-methionine cycle I	13
PWY-6612	superpathway of tetrahydrofolate biosynthesis	13
PWY-702	methionine biosynthesis II	13
PWY-801	homocysteine and cysteine interconversion	13
SUCUTIL-PWY	sucrose degradation I	13
PWY0-1182	trehalose degradation II (trehalase)	12
PWY2OL-4	linalool biosynthesis	12
PWY-5670	epoxysqualene biosynthesis	12
PWY-5988	wound-induced proteolysis I	12
PWY-6018	seed germination protein turnover	12
PWY-6554	1D-myo-inositol hexakisphosphate biosynthesis V (from Ins(1,3,4)P3)	12
BSUBPOLYAMSYN-PWY	spermidine biosynthesis I	11
COA-PWY	coenzyme A biosynthesis	11
GLUCOSE1PMETAB-PWY	glucose and glucose-1-phosphate degradation	11
GLUTSYNIII-PWY	glutamate biosynthesis III	11
POLYISOPRENSYN-PWY	polyisoprenoid biosynthesis (E. coli)	11

PWY-2781	cis-zeatin biosynthesis	11
PWY-5032	ent-kaurene biosynthesis I	11
PWY-5070	gibberellin biosynthesis I (non C-3, non C-13 hydroxylation)	11
PWY-6039	chlorogenic acid biosynthesis I	11
PWY-6823	molybdenum cofactor biosynthesis	11
PWY-6887	kauralexin biosynthesis	11
PWY-6908	thiamin diphosphate biosynthesis IV (eukaryotes)	11
PWY-881	trehalose biosynthesis II	11
PWY-882	ascorbate biosynthesis I (L-galactose pathway)	11
TREHALOSESYN-PWY	trehalose biosynthesis III	11
VALSYN-PWY	valine biosynthesis	11
ALACAT2-PWY	alanine degradation II (to D-lactate)	10
ARGININE-SYN4-PWY	arginine biosynthesis IV	10
ASPARTATE-DEG1-PWY	aspartate degradation I	10
ASPARTATESYN-PWY	aspartate biosynthesis	10
CYSTSYN-PWY	cysteine biosynthesis I	10
LEU-DEG2-PWY	leucine degradation I	10
METHIONINE-DEG1-PWY	methionine degradation I (to homocysteine)	10
NONOXIPENT-PWY	pentose phosphate pathway (non-oxidative branch)	10
PWY-101	photosynthesis light reactions	10
PWY-3385	choline biosynthesis I	10
PWY-3861	mannitol degradation II	10
PWY-6118	glycerol-3-phosphate shuttle	10
PWY-6515	phloridzin biosynthesis	10
PWY-6801	volatile esters biosynthesis (during fruit ripening)	10
PWY-922	mevalonate pathway I	10
PANTO-PWY	phosphopantothenate biosynthesis I	9
PWY-1822	IAA biosynthesis II	9
PWY-2902	cytokinins-O-glucoside biosynthesis	9
PWY-4041	gamma-glutamyl cycle	9
PWY-5084	2-ketoglutarate dehydrogenase complex	9
PWY-5669	phosphatidylethanolamine biosynthesis I	9
PWY-5725	farnesene biosynthesis	9
PWY-5905	hypusine biosynthesis	9
PWY-5963	thio-molybdenum cofactor biosynthesis	9
PWY-6122	5-aminoimidazole ribonucleotide biosynthesis II	9
PWY-6275	beta-caryophyllene biosynthesis	9
PWY-6365	D-myo-inositol (3,4,5,6)-tetrakisphosphate biosynthesis	9
PWY-6443	benzoate biosynthesis I (CoA-dependent, beta-oxidative)	9
PWY-6888	zealexin biosynthesis	9
PWY-6922	L-Ndelta-acetylornithine biosynthesis	9
PWY-6950	DIMBOA-glucoside biosynthesis	9
SO4ASSIM-PWY	sulfate reduction I (assimilatory)	9
UDPNACETYLGALSYN-PWY	UDP-N-acetyl-D-glucosamine biosynthesis II	9
GLUTAMINDEG-PWY	glutamine degradation I	8
PWY0-1314	fructose degradation	8
PWY3O-450	phosphatidylcholine biosynthesis I	8
PWY-4341	glutamate biosynthesis V	8
PWY-5098	chlorophyll a degradation I	8
PWY-5659	GDP-mannose biosynthesis	8
PWY-5886	4-hydroxyphenylpyruvate biosynthesis	8
PWY-6147	6-hydroxymethyl-dihydropterin diphosphate biosynthesis I	8
PWY-6368	3-phosphoinositide degradation	8

PWY-6927	chlorophyll a degradation II	8
CHOLINE-BETAINE-ANA-PWY	choline degradation I	7
GLUGLNSYN-PWY	glutamate biosynthesis IV	7
GLUTAMINEFUM-PWY	glutamine degradation II	7
GLUTSYN-PWY	glutamate biosynthesis I	7
MENAQUINONESYN-PWY	menaquinol-8 biosynthesis	7
PWY0-1275	lipoate biosynthesis and incorporation II	7
PWY0-1301	melibiose degradation	7
PWY0-662	PRPP biosynthesis I	7
PWY-3461	tyrosine biosynthesis II	7
PWY-4321	glutamate degradation IV	7
PWY-4702	phytate degradation I	7
PWY-4821	UDP-D-xylose and UDP-D-glucuronate biosynthesis	7
PWY-4984	urea cycle	7
PWY-5148	acyl-CoA hydrolysis	7
PWY-5188	tetrapyrrole biosynthesis I	7
PWY-5849	menaquinol-6 biosynthesis	7
PWY-6120	tyrosine biosynthesis III	7
PWY-6163	chorismate biosynthesis from 3-dehydroquinate	7
PWY-6717	(1,4)-beta-xylan degradation	7
SAM-PWY	S-adenosyl-L-methionine biosynthesis	7
XYLCAT-PWY	xylose degradation I	7
GLUTAMATE-DEG1-PWY	glutamate degradation I	6
GLUT-REDOX-PWY	glutathione redox reactions II	6
P21-PWY	pentose phosphate pathway (partial)	6
PLPSAL-PWY	pyridoxal 5'-phosphate salvage pathway	6
PWY0-1305	glutamate dependent acid resistance	6
PWY1F-353	glycine betaine biosynthesis III (plants)	6
PWY3O-210	glutamate degradation IX (via 4-aminobutyrate)	6
PWY3O-440	acetoin biosynthesis III	6
PWY-4261	glycerol degradation I	6
PWY-5111	CMP-KDO biosynthesis II (from D-arabinose 5-phosphate)	6
PWY-5123	trans, trans-farnesyl diphosphate biosynthesis	6
PWY-5194	siroheme biosynthesis	6
PWY-5337	stachyose biosynthesis	6
PWY-5441	S-methylmethionine cycle	6
PWY-5852	demethylmenaquinol-8 biosynthesis I	6
PWY-5872	ubiquinol-10 biosynthesis (eukaryotic)	6
PWY-5945	antheraxanthin and violaxanthin biosynthesis	6
PWY-6330	acetaldehyde biosynthesis II	6
PWY-6481	L-dopachrome biosynthesis	6
PWY-6710	poly-hydroxy fatty acids biosynthesis	6
PWY-6752	o-diquinones biosynthesis	6
PWY-695	abscisic acid biosynthesis	6
PROUT-PWY	proline degradation	5
PWY0-501	lipoate biosynthesis and incorporation I	5
PWY0-522	lipoate salvage and modification	5
PWY-1422	vitamin E biosynthesis	5
PWY-2301	myo-inositol biosynthesis	5
PWY-4861	UDP-D-galacturonate biosynthesis I (from UDP-D-glucuronate)	5
PWY-5068	chlorophyll cycle	5
PWY-5097	lysine biosynthesis VI	5

PWY-5122	geranyl diphosphate biosynthesis	5
PWY-5129	sphingolipid biosynthesis (plants)	5
PWY-5474	hydroxycinnamic acid tyramine amides biosynthesis	5
PWY-5806	all-trans-decaprenyl diphosphate biosynthesis	5
PWY-6458	benzoyl-CoA biosynthesis	5
PWY-6802	salidroside biosynthesis	5
SERSYN-PWY	serine biosynthesis	5
ARG-PRO-PWY	arginine degradation VI (arginase 2 pathway)	4
GLUTAMATE-SYN2-PWY	glutamate biosynthesis II	4
GLYCLEAV-PWY	glycine cleavage complex	4
PWY0-1021	alanine biosynthesis III	4
PWY0-1264	biotin-carboxyl carrier protein assembly	4
PWY0-1507	biotin biosynthesis from 7-keto-8-aminopelargonate	4
PWY-102	gibberellin inactivation I (2beta-hydroxylation)	4
PWY-3982	uracil degradation II (reductive)	4
PWY-4781	phytate degradation II	4
PWY-4983	citrulline-nitric oxide cycle	4
PWY4FS-6	phosphatidylethanolamine biosynthesis II	4
PWY-5034	GA12 biosynthesis	4
PWY-5046	branched-chain alpha-keto acid dehydrogenase complex	4
PWY-5074	mevalonate degradation	4
PWY-5142	acyl-ACP thioesterase pathway	4
PWY-5278	sulfite oxidation III	4
PWY-5340	sulfate activation for sulfonation	4
PWY-5766	glutamate degradation X	4
PWY-5785	di-trans,poly-cis-undecaprenyl phosphate biosynthesis	4
PWY-5885	wax esters biosynthesis II	4
PWY-6012	acyl carrier protein metabolism	4
PWY-6281	selenocysteine biosynthesis II (archaea and eukaryotes)	4
PWY-6299	aldehyde oxidation I	4
PWY-6358	superpathway of D-myo-inositol (1,4,5)-trisphosphate metabolism	4
PWY-6364	D-myo-inositol (1,3,4)-trisphosphate biosynthesis	4
PWY-6556	pyrimidine ribonucleosides degradation II	4
PWY-6745	phytochelatins biosynthesis	4
PYRIDNUCSYN-PWY	NAD biosynthesis I (from aspartate)	4
SULFMETII-PWY	sulfate reduction II (assimilatory)	4
THIOREDOX-PWY	thioredoxin pathway	4
ASPARAGINE-DEG1-PWY	asparagine degradation I	3
GLYSYN-THR-PWY	glycine biosynthesis IV	3
HOMOSERSYN-PWY	homoserine biosynthesis	3
PWY1F-467	phenylpropanoid biosynthesis, initial reactions	3
PWY-3341	proline biosynthesis III	3
PWY3DJ-11281	sphingomyelin metabolism	3
PWY-4361	methionine salvage I (bacteria and plants)	3
PWY-4981	proline biosynthesis II (from arginine)	3
PWY-5035	gibberellin biosynthesis III (early C-13 hydroxylation)	3
PWY-5120	geranylgeranyldiphosphate biosynthesis	3
PWY-5153	anthocyanin biosynthesis (delphinidin 3-O-glucoside)	3
PWY-5172	acetyl-CoA biosynthesis III (from citrate)	3
PWY-5674	nitrate reduction IV (dissimilatory)	3
PWY-5704	urea degradation II	3
PWY-5874	heme degradation	3
PWY-6019	pseudouridine degradation	3

PWY-6029	2,3-trans-flavanols biosynthesis	3
PWY-6124	inosine-5'-phosphate biosynthesis II	3
PWY-6363	D-myo-inositol (1,4,5)-trisphosphate degradation	3
PWY-641	proanthocyanidin biosynthesis from flavanols	3
PWY-6543	p-aminobenzoate biosynthesis	3
PWY-6653	ent -kaurene biosynthesis II	3
PWY-6898	thiamin salvage III	3
ALANINE-SYN2-PWY	alanine biosynthesis II	2
GLUCONSUPER-PWY	D-gluconate degradation	2
GLYCEROLMETAB-PWY	glycerol degradation V	2
HOMOSER-THRESYN-PWY	threonine biosynthesis from homoserine	2
LIPAS-PWY	triacylglycerol degradation	2
MANNCAT-PWY	D-mannose degradation	2
PWY0-461	lysine degradation I	2
PWY-2181	free phenylpropanoid acid biosynthesis	2
PWY-3041	monoterpene biosynthesis	2
PWY4FS-7	phosphatidylglycerol biosynthesis I (plastidic)	2
PWY4FS-8	phosphatidylglycerol biosynthesis II (non-plastidic)	2
PWY-5086	chlorophyll a biosynthesis I	2
PWY-5138	fatty acid beta-oxidation IV (unsaturated, even number)	2
PWY-5175	lactucaxanthin biosynthesis	2
PWY-5269	cardiolipin biosynthesis II	2
PWY-5271	phaseic acid biosynthesis	2
PWY-5343	ajugose biosynthesis II (galactinol-independent)	2
PWY-5691	urate degradation to allantoin	2
PWY-5936	xyloglucan biosynthesis	2
PWY-5946	delta-carotene biosynthesis	2
PWY-6326	camptothecin biosynthesis	2
PWY-6366	D-myo-inositol (1,4,5,6)-tetrakisphosphate biosynthesis	2
PWY-6466	pyridoxal 5'-phosphate biosynthesis II	2
PWY-6482	diphthamide biosynthesis	2
PWY-6599	guanine and guanosine salvage II	2
PWY-6605	adenine and adenosine salvage II	2
PWY-6614	tetrahydrofolate biosynthesis	2
PWY-6689	tRNA splicing	2
PWY-66	GDP-L-fucose biosynthesis I (from GDP-D-mannose)	2
PWY-6755	S-methyl-5-thio-alpha-D-ribose 1-phosphate degradation	2
PWY-723	alkylnitronates degradation	2
CITRULLINE-DEG-PWY	citrulline degradation	1
GLUTATHIONESYN-PWY	glutathione biosynthesis	1
GLYSYN-ALA-PWY	glycine biosynthesis III	1
PWY-3221	dTDP-L-rhamnose biosynthesis II	1
PWY-401	glycolipid biosynthesis	1
PWY-4841	UDP-D-glucuronate biosynthesis (from myo-inositol)	1
PWY-4921	protein citrullination	1
PWY-5045	pinosylvin metabolism	1
PWY-5203	soybean saponin I biosynthesis	1
PWY-5276	sulfite oxidation I (sulfite oxidoreductase)	1
PWY-5326	sulfite oxidation IV	1
PWY-5350	thiosulfate disproportionation III (rhodanese)	1
PWY-5377	alpha-amyrin biosynthesis	1
PWY-5403	betaxanthin biosynthesis (via dopamine)	1
PWY-5426	betaxanthin biosynthesis	1
PWY-5434	(3E)-4,8-dimethylnona-1,3,7-triene biosynthesis	1

PWY-5481	pyruvate fermentation to lactate	1
PWY-5783	octaprenyl diphosphate biosynthesis	1
PWY-5805	nonaprenyl diphosphate biosynthesis I	1
PWY-5871	ubiquinol-9 biosynthesis (eukaryotic)	1
PWY-5943	beta-carotene biosynthesis	1
PWY-5944	zeaxanthin biosynthesis	1
PWY-5957	nicotianamine biosynthesis	1
PWY-6173	histamine biosynthesis	1
PWY-6199	quercetinsulphates biosynthesis	1
PWY-6273	phosphatidylethanolamine biosynthesis III	1
PWY-6323	benzoylanthranilate biosynthesis	1
PWY-63	UDP-L-arabinose biosynthesis I (from UDP-xylose)	1
PWY-6440	spermine and spermidine degradation II	1
PWY-6441	spermine and spermidine degradation III	1
PWY-6457	trans-cinnamoyl-CoA biosynthesis	1
PWY-6473	4-aminobutyrate degradation IV	1
PWY-6520	nonaprenyl diphosphate biosynthesis II	1
PWY-6539	petivericin biosynthesis	1
PWY-6619	adenine and adenosine salvage VI	1
PWY-6754	S-methyl-5'-thioadenosine degradation I	1
PWY-6799	fatty acid biosynthesis (plant mitochondria)	1
PWY-6825	phosphatidylcholine biosynthesis V	1
PWY-6939	tetrahydroxystilbenes biosynthesis	1
PWY-6	GDP-L-fucose biosynthesis II (from L-fucose)	1
PWY-82	UDP-L-arabinose biosynthesis II (from L-arabinose)	1
PWY-84	resveratrol biosynthesis	1

Supplementary Table S4. Correlation of spinach accessions.

sample	Sp78	Sp82	Sp90	Sp49	Sp50	Sp51	Sp42	Sp43	Sp40
Sp78	1	0.984	0.962	0.897	0.903	0.953	0.424	0.387	0.931
Sp82	0.984	1	0.957	0.897	0.894	0.952	0.411	0.378	0.919
Sp90	0.962	0.957	1	0.883	0.922	0.98	0.406	0.371	0.935
Sp49	0.897	0.897	0.883	1	0.917	0.91	0.381	0.297	0.923
Sp50	0.903	0.894	0.922	0.917	1	0.943	0.443	0.377	0.967
Sp51	0.953	0.952	0.98	0.91	0.943	1	0.412	0.355	0.943
Sp42	0.424	0.411	0.406	0.381	0.443	0.412	1	0.913	0.444
Sp43	0.387	0.378	0.371	0.297	0.377	0.355	0.913	1	0.367
Sp40	0.931	0.919	0.935	0.923	0.967	0.943	0.444	0.367	1
